# Effect of DMPA and Molecular Weight of Polyethylene Glycol on Water-Soluble Polyurethane

**DOI:** 10.3390/polym11121915

**Published:** 2019-11-21

**Authors:** Eyob Wondu, Hyun Woo Oh, Jooheon Kim

**Affiliations:** School of Chemical Engineering and Materials Science, Chung-Ang University, Seoul 156-756, Korea; wendueyoba@gmail.com (E.W.); ohwoo7@naiver.com (H.W.O.)

**Keywords:** water-soluble polyurethane, molecular weight, thermal properties, contact angle

## Abstract

In this study water-soluble polyurethane (WSPU) was synthesized from isophorone diisocyanate (IPDI), and polyethylene glycol (PEG), 2-bis(hydroxymethyl) propionic acid or dimethylolpropionic acid (DMPA), butane-1,4-diol (BD), and triethylamine (TEA) using an acetone process. The water solubility was investigated by solubilizing the polymer in water and measuring the contact angle and the results indicated that water solubility and contact angle tendency were increased as the molecular weight of the soft segment decreased, the amount of emulsifier was increased, and soft segment to hard segment ratio was lower. The contact angle of samples without emulsifier was greater than 87°, while that of with emulsifier was less than 67°, indicating a shift from highly hydrophobic to hydrophilic. The WSPU was also analyzed using Fourier transform infrared spectroscopy (FT-IR) to identify the absorption of functional groups and further checked by X-ray photoelectron spectroscopy (XPS). The molecular weight of WSPU was measured using size-exclusion chromatography (SEC). The structure of the WSPU was confirmed by nuclear magnetic resonance spectroscopy (NMR). The thermal properties of WSPU were analyzed using thermogravimetric analysis (TGA), and differential scanning calorimetry (DSC).

## 1. Introduction

A need for water-soluble polyurethane arises as it is advantageous for adhesion to various dispersions, resistance to chemicals, solvents, and water, resistance to abrasion, high flexibility and toughness, non-flammability, easy water cleanup product, low volatile organic compounds (VOC) less pollution and fast dry products increase [1,2,3,4,5,6,7,8,9].

To ensure water solubility of polyurethane (PUR), the PUR’s molecular weight and viscosity should not be too high, and the concentration of polar groups (carboxylic groups, sulfuric acid groups, and tertiary amine groups) must be high enough. Water-soluble polyurethanes (WSPU)s can be divided into two classes of polymer stabilization: (1) external emulsifiers and (2) hydrophilic centers in the polymer (nonionic groups like polyethylene oxide chains, cationic groups as alkylated or protonated tertiary amines and anionic groups including carboxylate or sulfonate groups) [1,3,4,10].

Polyurethanes are prepared using a low to medium molecular weight isocyanate prepolymer from di or polyols with di- or poly-isocyanates, followed by chain extending and dispersing the prepolymer in water by introducing hydrophilic solubility groups [10,11,12,13]. There are various raw materials for WSPU production: polyols (OH), diisocyanate (DI) (either aromatic or aliphatic), chain extenders, neutralizers, catalysts, and solvents. OH and DI are the most important, being the large constituents of soft segments and hard segments of the WSPU, respectively. WSPUs produced from aromatic DI degrade easily in ultraviolet (UV) radiation, while the aliphatic DI-based WSPUs are UV-resistant [10,14,15,16]. Polyethers, polyesters, polydienes, and polyolefins are the OHs most widely used to synthesize WSPUs [14].

WSPU is low in volatile organic compounds (VOCs), which allows it to be used in coatings for various fibers, paint additives, primers for metals, associated thickeners, caulking materials, adhesives for alternative substrates, emulsion polymerization media for different monomers, pigment pastes, defoamers, and textile dyes [4,12,14,17,18,19,20]. Depending on the starting components, solvents, and process sequence, several preparation methods are common, such as acetone, prepolymer mixing, melt process, and ketamine–ketazine process [2,4,7,12,14,15,17,18,20,21,22,23,24]. In the acetone process, excess viscosity is prevented using the solvent as chain extension agent, in the prepolymer process the PUR prepolymer is chain extended using polyamine or diamine during the dispersion stage. During the melt process an oligomer is formed by reacting a low-viscosity isocyanate prepolymer ionically/nonionically modified with either ammonia or urea. The ketamine–ketazine process is aqueous phase dispersion of blends of a blocked amine (ketamine) or hydrazine (ketazine) isocyanate prepolymer [8,10,14,25,26,27].

WSPUs can be anionic, cationic, or nonionically stabilized. Anionic WSPU stabilization is the most common process today; examples include functionalized co-monomers of sulfonate or carboxylate, dihydroxy sulfonic acids, and dimethylolpropionic acids (DMPA) [8,10].

WSPUs are classified based on two characteristics according to Szycher’s: internal stabilization mechanism (nonionic, anionic, and cationic), which also defines the pH range to be used, and chemical composition (the type of polyisocyanates, either aromatic or aliphatic; polyol groups, polyester, polyether, or polycarbonate) used in manufacturing the WSPU, which defines characteristics of the final product [10].

Aliphatic diisocyanates or polyisocyanates include isophorone diisocyanate (IPDI), hexamethylene diisocyanate (HDI), 4,4-methylenebis (cyclohexyl isocyanate) (HMDI), and tetramethylxylene diisocyanate (TMXDI), and are used to resist yellowing and UV degradation. Conversely, aromatic diisocyanate or polyisocyanates include toluene diisocyanate (TDI), 4,4′-Methylenebis(phenyl isocyanate) (MDI), and polymeric grades, and are applicable in areas where yellowing is tolerable and UV radiation is negligible or acceptable [10].

In this study, anionic stabilized WSPUs were produced via the acetone process, due to the acetone processes easy processability, low cost, and preventing excess viscosity, using dimethylolpropionic acid (DMPA) as an emulsifier and aliphatic diisocyanates with polyether diols, polyethylene glycol (PEG), and triethylamine (TEA) as neutralizer, and 1,4-butanediol (BD) as chain extender. The effect of the variation of molecular weight of PEG and the influence of DMPA content on the water solubility of WSPU was studied.

## 2. Materials and Methods

### 2.1. Materials

Isophorone diisocyanate (98%, IPDI), dibutyltin dilaurate (99%, DBTDL), dimethylolpropionic acid (98%, DMPA), triethylamine (≥99%, TEA), and 1,4-butane diol (99%, BD) were obtained from Sigma-Aldrich (St. Louis, MO, USA). Polyethylene glycol 1500 (PEG1500), polyethylene glycol 4000 (PEG4000), polyethylene glycol 8000 (PEG8000), acetone, and hexane were received from Dae-Jung Chemical and Metal Co. LTD (Seoul, Korea). All chemicals were used as received.

### 2.2. Methods of WSPU Production

The acetone process was used to prepare the WSPU [12]. PEG:IPDI at different ratios of 1:1, 1:6, and 1:9 was charged to the acetone-containing reactor, and 2 and 4% DMPA was added to the solvent-containing reactor for each ratio. The reactor was stirred using a magnetic stirrer in an oil bath until the solution became homogeneous. Once the solution was homogenized, a suitable amount of IPDI (according to the molar ratio) and 3 drops of DBTDL were added to the reactor. The reaction occurred at 60 °C. The reaction proceeded for 6 h; then, chain extender BD (0.6%) was charged and the reaction was left to proceed at the same temperature for additional 2 h. Then, the temperature was decreased to 50 °C and appropriate amount of neutralizer (equimolar amount of DMPA), triethylamine, was added to the system and stirred vigorously for 30 min. After neutralization, the products were precipitated in a hexane-containing beaker, and the catalyst and acetone remained with the hexane while the polyurethane precipitated at the bottom. The precipitate was dried for about 3 days in free air, and the resulting film was used for further analysis. All percentages used here are weight percentages (*wt*/*wt*) and the ratios are molar.

### 2.3. Characterizations

Fourier transform infrared spectroscopy (FT-IR; Nicolet, is5, Thermo Fisher Scientific, Seoul, Korea) and X-ray photoelectron spectroscopy (XPS; K-Alpha, Thermo Fisher Scientific) were used to compare the infrared spectra of each sample with the PEG samples. A background of KBr (potassium bromide) powder was utilized during FT-IR analysis and a frequency range of 4000–400 cm^−1^ was used to record the data. A step size of 1.0 eV and a survey pass energy of 200 eV were used during XPS measurements. Background for the XPS measurement was monochromatic Al Kα, and the results were curve-fitted, with all Gaussian-fitted width peaks of the spectrum held constant. The chemical structure of the polymer was confirmed by using nuclear magnetic resonance spectroscopy (^1^HNMR 300 Hz, Gemini 2000, Varian, Palo Alto, CA, USA), using dimethyl sulfoxide-d_6_ (DMSO) as a reference after solubilizing the samples in a deuterated solvent, chloroform (CDCl_3_).

WSPUs molecular weight was determined by size-exclusion chromatography (SEC) using Waters HPLC (Alliance 2695, UV/Vis Detector 2489, Waters corporation, Milford, MA, USA) with a dual detector of UV 220/280 nm. The separation was carried out at 30 °C column temperature, Biosuite high resolution SEC column (250 A, 10 µm, 7.5 × 300 mm^2^). Mobile phase of 150 mM sodium phosphate, pH 7 was used. The specimens were prepared by dissolving WSPU samples in acetone. Water/methanol mixture was used as a seal wash (90%/10% (*v*/*v*)).

The thermal stability of the samples with and without DMPA and chain elongation were analyzed using thermogravimetric analysis (TGA-2050, TA Instruments, New Castle, DE, USA). Approximately 10 mg of each sample was heated to 600 °C at a heating rate of 10 °C/min to check the thermal degradation in a nitrogen atmosphere. Differential scanning calorimetry (DSC-EVO, KEP Tech., Mougins, France) was used to measure the melting temperature and melting enthalpy of the obtained polymer. The specimens were cooled down through quenching using liquid nitrogen to −60 °C and heated up to 200 °C with a heating rate of 10 °C/min.

The hydrophobic nature of all the specimens with 4% DMPA and with no DMPA was measured by a drop shape analyzer (DSA100, KRÜSS GmbH, Hamburg, Germany) at room temperature. The samples were hot-pressed to form a flat surface at 60 °C for about 20 min, a single sample was measured at least five times, and the average value was taken as a contact angle of a single specimen using the sessile drop method with a drop of 5 μL of water.

This section ahead the abbreviations 1500PEG, 4000PEG, 8000PEG, PUR, and PEG: IPDI, imply that 1500 molecular weight polyethylene glycol, 4000 molecular weight polyethylene glycol, 8000 molecular weight polyethylene glycol, polyurethane, and the ratio of polyethylene glycol to isophorone diisocyanate, respectively. The ratios are molar ratios and the percentages are weight percentages.

## 3. Results and Discussions

**Figure 1:**
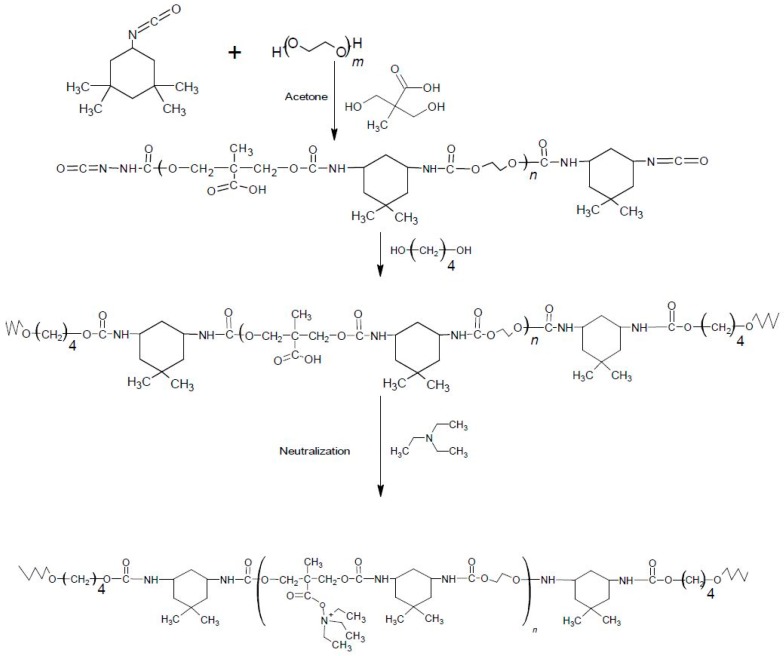
Reaction scheme of water-soluble polyurethane (WSPU) production.

Aliphatic-based isocyanate, 1,4-butanediol, dimethylolpropionic acid, triethylamine, and polyethylene glycols of number average molecular weights of 1500, 4000, and 8000 were used to produce the WSPU. The WSPUs were made using the acetone process in an oil bath. The samples with DMPA and without DMPA were prepared and analyzed by comparison. The DMPA content was varied to analyze its effect on the water solubility of the PUR. Figure 1 shows the reaction scheme of WSPU production using the acetone process.

**Figure 2:**
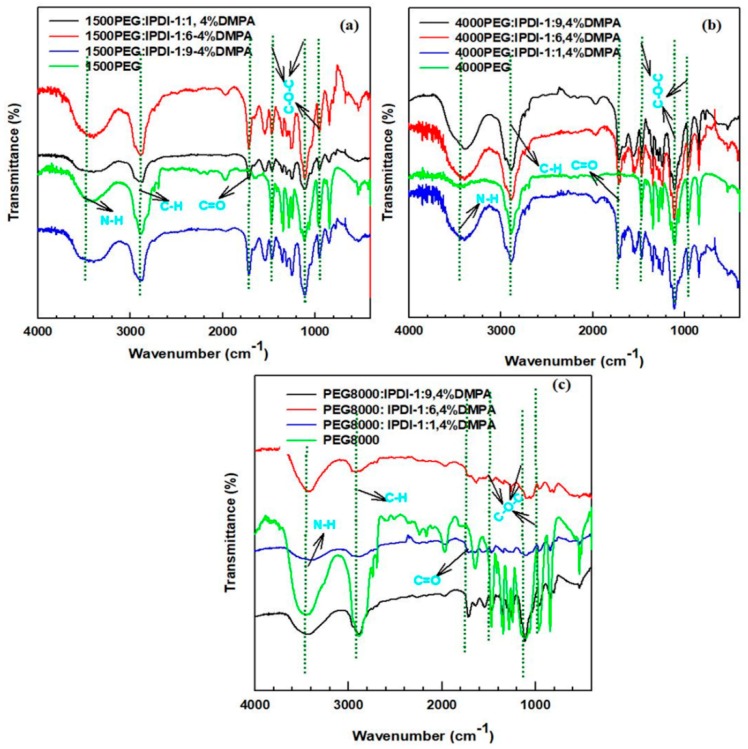
FT-IR results of WSPU: (**a**) 1500 polyethylene glycol (PEG): isophorone diisocyanate (IPDI) with 4% dimethylolpropionic acid (DMPA) and 1500PEG, (**b**) 4000PEG: IPDI with 4% DMPA and 4000PEG, (**c**) 8000PEG:IPDI with 4% DMPA and 8000PEG.

The FT-IR of the WSPU is shown in Figure 2. The NH-stretching peaks prove the appearance of the PUR structure. The polyurethane structure is proved by noting the absorption band in between 3200–3500 cm^−1^ (N–H stretching, urethane linkage). This peak intensity increased with an increase in the hard segment content, indicating the formation of more –CO–NH– linkages. Free vibrating frequencies of the carbonyl regions appeared at a wavenumber near 1726 cm^−1^. The intensity of the band attributed to free and hydrogen-bonded urethane carbonyls (1726 and 1708 cm^−1^) increased with an increase in the hard segment content of PUR because of the increasing formation of urethanes bonds. As compared to the 8000PEG, the data show that intensity of the peaks of the N–H group increased and a new peak appeared at 1720 cm^−1^, which corresponds to the C=O functional groups from IPDI and DMPA. The increase in the intensity of the N–H peak is due to the addition of an amine group containing IPDI, with triethylamine as a neutralizer. As the hard segment content increases, the intensity of the N–H peak increases.

**Figure 3:**
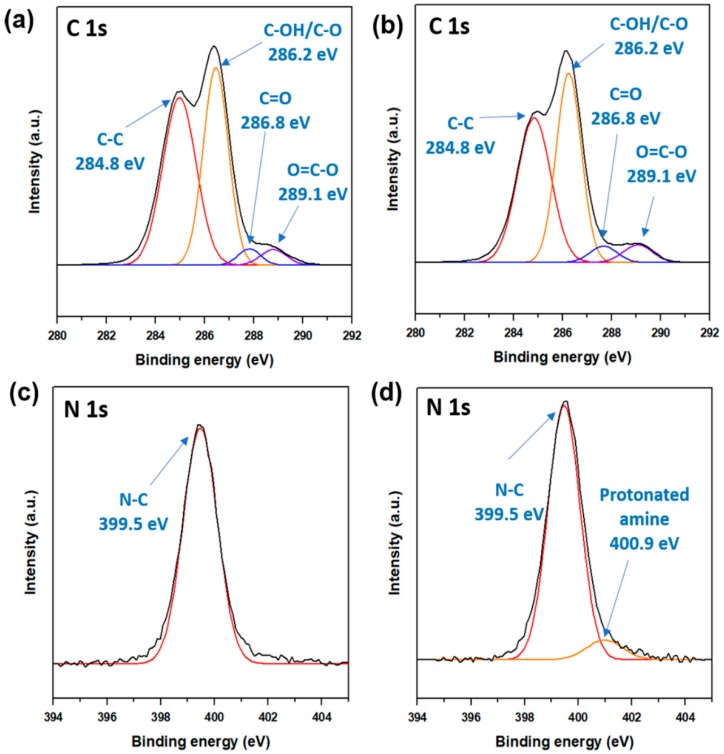
X-ray photoelectron spectroscopy (XPS) deconvolution results of WSPU: (**a**) C1s of WSPU without DMPA, (**b**) C1s of WSPU with 4% DMPA, (**c**) N1s of WSPU without DMPA and (**d**) N1s of WSPU with 4% DMPA.

The results of XPS deconvolution is shown in Figure 3. C1s peaks that correspond to aliphatic carbons were observed at 284.8 eV. The C1s peaks at 286.2 and 286.8 eV were due to amino and ethereal carbons, respectively. C1s peaks of urethane groups, ester, and carboxylic acid were observed at 289.1 eV. The one with DMPA (right side) had high intensity in terms of C1s peaks as compared to the one without DMPA (left side), as shown in Figure 3a. The N1s peaks that correspond to amine and amide groups were observed at 401.5 and 399.5, respectively, both with DMPA and without DMPA.

**Figure 4:**
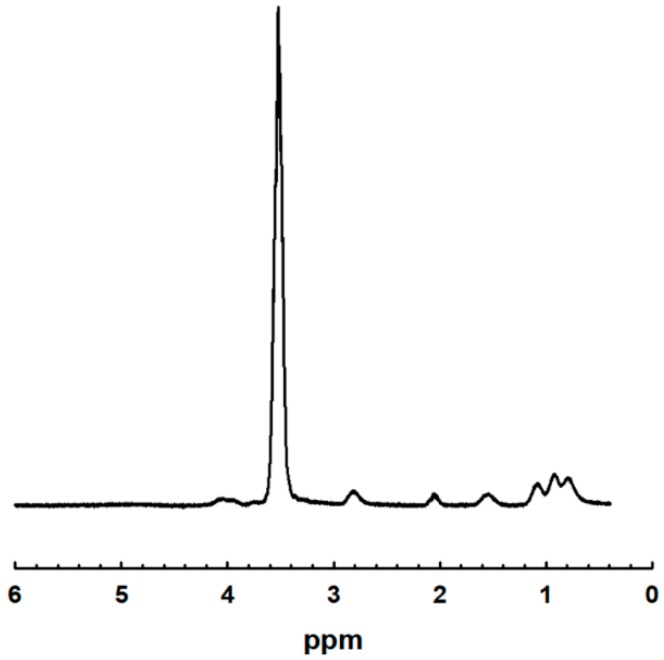
NMR structure of WSPU.

^1^HNMR spectroscopy was used to verify the chemical structure of WSPU. The proton NMR signals were done using CDCl_3_ as solvents, shown in Figure 4, in which the structure of WSPU is confirmed. The longest signal, appearing at 3.64 ppm, was due to the repeated methylene units in PEG components, while that of the IPDI components is not visible due to the low fractions used, but it appeared around 3.8 ppm in small amounts. Rather the structure of the remaining components, that of DMPA, BD, and TEA was not observed because the low fraction of the components used while producing the WSPU. The number of protons were different for each samples, found from integration of the splitting results, depending on the molecular weight of PEG used and is shown in Table 1.

**Figure 5:**
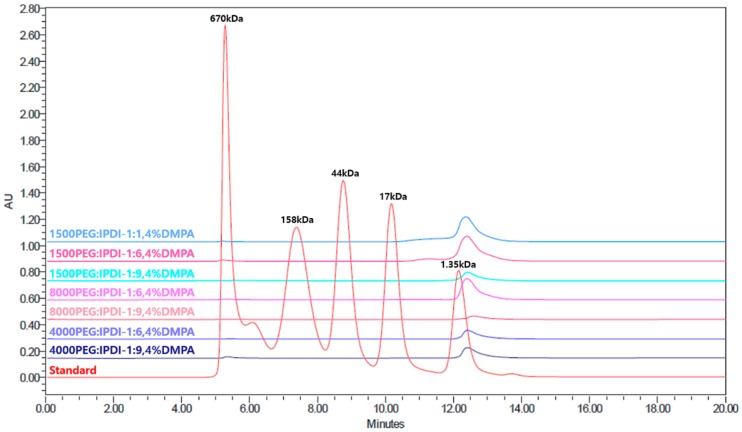
Size-exclusion chromatography (SEC) analysis results of molecular weight determination.However, the results of SEC analysis revealed that the molecular weights were almost equal regardless of the variation in molecular weight of PEG and PEG: PDI ratio. It was expected the molecular weight would increase as the PEG molecular weight increased and PEG: IPDI ratio increased (hard segment content increased) [28]. Nevertheless, this does not apply for all process systems [29]. Diez-Garcia et al. has got results with decreasing molecular weight of WSPU as PEG content increased in contrast to our results which were constant [30].

The molecular weight of the synthesized WSPU is determined by SEC, shown in Table 1. The molecular weight of seven samples with various molecular weight of PEG and different PEG:IPDI ratios are measured. The SEC analysis results are shown in Figure 5.

**Figure 6:**
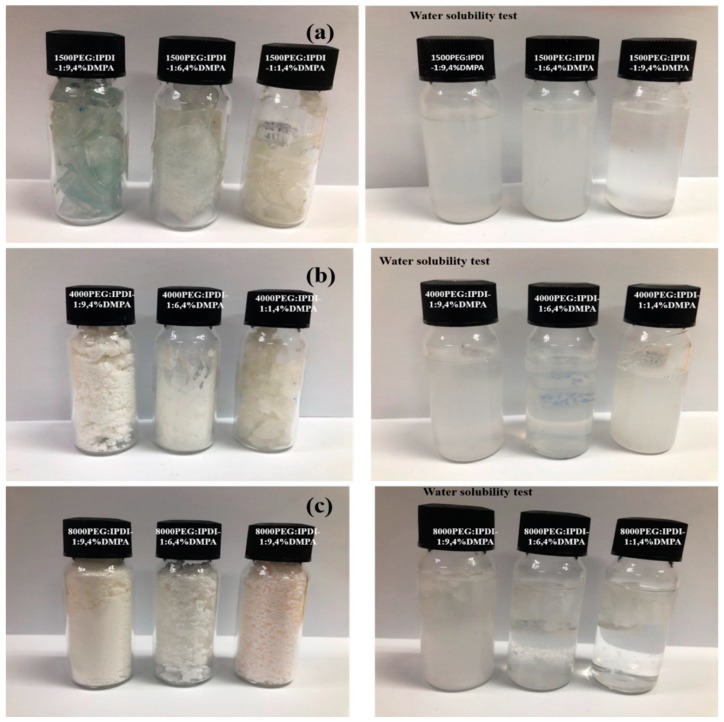
Water solubility test results: (**a**) 1500PEG:IPDI 1:9, 4% DMPA, (**b**) 4000PEG:IPDI 1:9, 4% DMPA, (**c**) 8000PEG:IPDI 1:9, 4% DMPA.

The water solubility test results for the WSPU samples are shown in Figure 6. On the left are WSPU powder samples, and on the right are WSPU samples dispersed in water. Figure 6a–c shows 1500 PEG:IPDI samples, 4000 PEG:IPDI samples, and 8000 PEG:IPDI samples respectively. The higher the molecular weight of PEG used in the formation of WSPU, the less the product was water-soluble; an increase in PEG molecular weight resulted in a decrease in water solubility. Furthermore, as the quantity of PEG increased (a higher PEG:IPDI ratio), both the molar concentration of PEG and the water solubility increased. The water solubility test results are shown in Table 1. Moreover, the solubility of the products increased with an increase in the DMPA content, as the solubility rate increased with an increase in DMPA content.

1500PEG-made WSPU completely soluble in water within a short period of time as compared to the 4000PEG-made WSPU, and that of 8000PEG. A comparison between the water solubility of WSPU synthesized from 2% DMPA and 4% DMPA with the same molecular weight of PEG depicts that the one with 4% DMPA had better solubility. For the same ratio of PEG:IPDI and same amount of DMPA but different molecular weight of PEG, the samples with less molecular weight were more soluble as compared to the corresponding counterparts with higher molecular weight of PEG.

**Figure 7:**
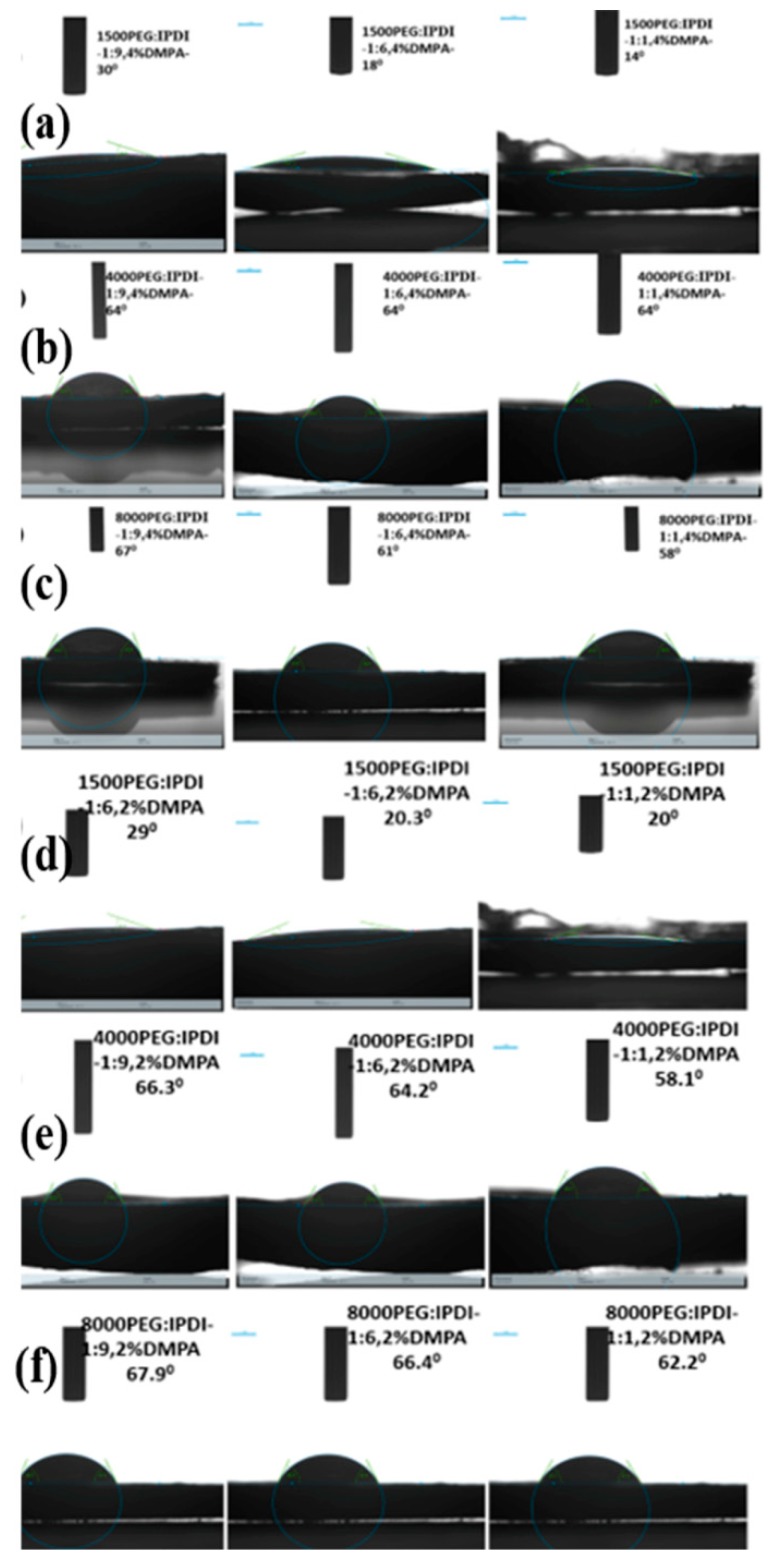
Contact angle of WSPU: (**a**) 1500 PEG: IPDI, 4% DMPA, (**b**) 4000 PEG: IPDI, 4% DMPA, (**c**) 8000 PEG: IPDI, 4% DMPA, (**d**) 1500 PEG: IPDI, 2% DMPA, (**e**) 4000 PEG: IPDI, 4% DMPA and (**f**) 8000 PEG: IPDI, 4% DMPA.

Measurement of contact angle between flat surface of WSPU film and water droplet was measured to analyze the hydrophilic nature of the product. Figure 7 shows the results of water contact angle of various WSPU samples. The results reveal that there was a difference in contact angle depending on the content of hard segment and molecular weight of PEG. Mostly, the contact angle value was affected by the molecular weight of PEG. As the molecular weight of PEG increased, the contact angle value increased and thus the hydrophilic nature of the compound decreased or the compound became more hydrophobic as compared to WSPU with low molecular weight PEG. Moreover, the hard segment content also affected the contact angle value but not as significant as the molecular weight of PEG did. The contact angle value increased as the hard segment content increased, meaning that the hydrophobicity had increased or its hydrophilic nature was hindered. This was due to the decrease in hydroxyl group bonds as the content of hard segment increased. In addition to this the rough, not flat, and non-uniform nature of the specimens contributed to the variation in the wetting property of the WSPU samples. The average value of contact angle was found by taking at least five data for one specimen and this is shown in Table 1. The contact angle decreased with an increase in DMPA content, as the emulsification ability increased with an increase in DMPA content.

Contact angle of WSPU with and without DMPA were also compared. The ones containing DMPA were hydrophilic and the measured contact angle were in ranges between 10°–70°, and the others synthesized without DMPA exhibit more hydrophobic nature with the contact angle lying between 87°–98°. Therefore, DMPA had given hydrophilic properties to the WSPU due to the presence of water-loving (hydrophilic) carboxylic groups in its chain.

**Figure 8:**
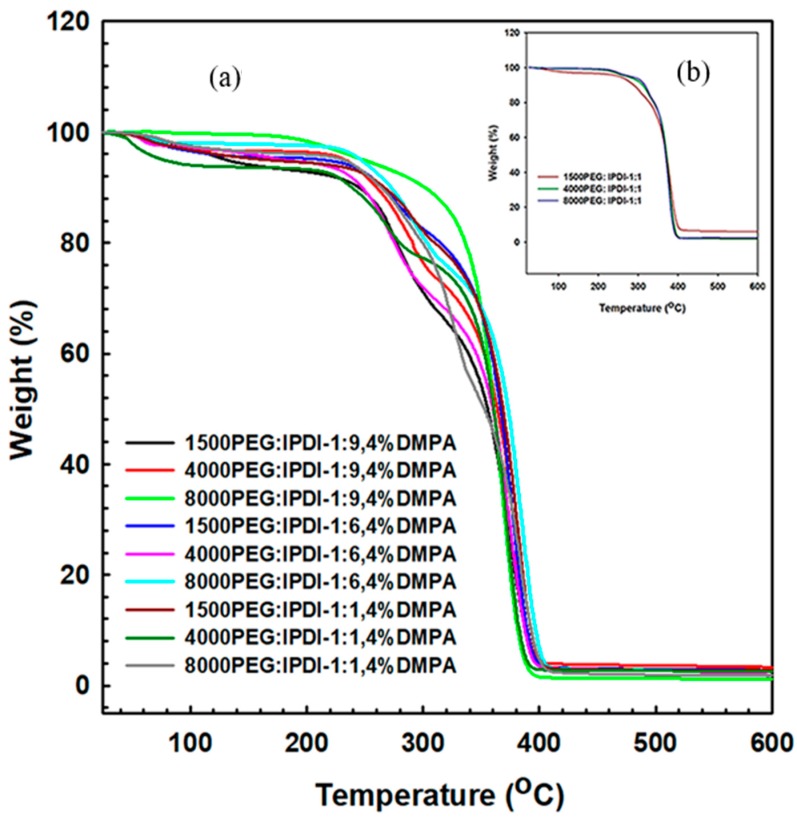
Thermal degradation properties of (**a**) WSPU (**b**) PUR without DMPA.

The thermal properties of WSPU, shown in Figure 8a, depended primarily on the polyol and isocyanate content. As the molecular weight of polyol increased, the thermal degradation temperature of WSPU increased, and vice versa. For the same molecular weight of polyol, as the IPDI content increased, the thermal degradation temperature increased. Thermal degradation occurred at various stages. The degradation of WSPU weight started at 230–250 °C and degraded more sharply at 310–350 °C. These weight losses at different temperatures were due to the decomposition of various urethane bonds. The variation in temperature was due to the molecular weight increase in polyol and the increase in the IPDI content of the WSPU. Figure 8b shows the thermal degradation behavior of PUR without DMPA, the trend of degradation was similar with the WSPU, an increase in molecular weight of polyol led to an increase in the thermal degradation temperature.

**Figure 9:**
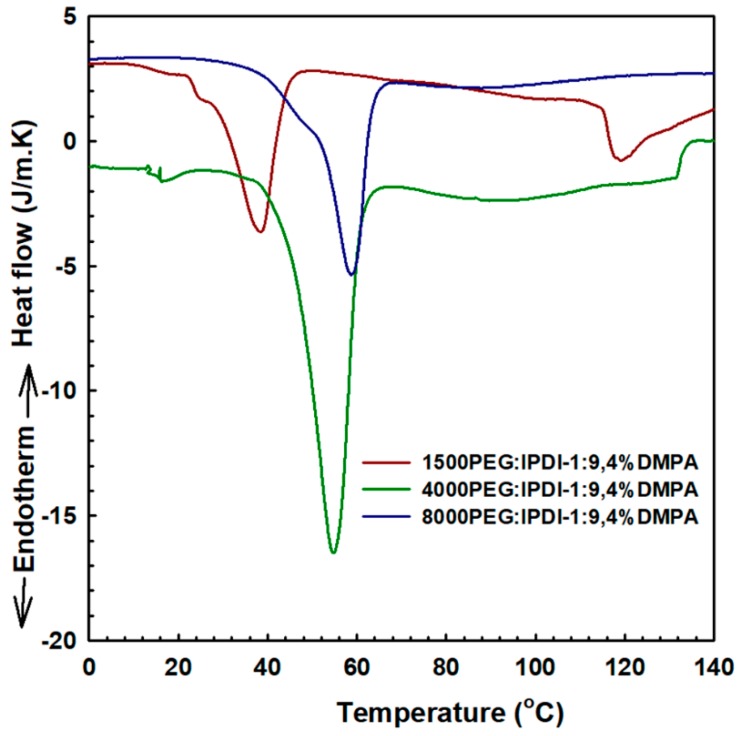
Thermophysical property of WSPU samples.

The other thermoanalytical technique used was differential scanning calorimetry (DSC), which measures the difference in the amount of heat required to increase the temperature of a given sample and reference as a function of temperature. The DSC results were shown in Figure 9 for the samples of 1500 PEG, 4000 PEG and 8000 PEG:IPDI-1:3,4%DMPA. The reference sample used in this study was a crucible made of aluminum (Al30). The melting peaks of the samples were analyzed and found to be in the same region, in between 45–63 °C, which corresponded to the melting point of the PEG components, soft segments, which was the highest fraction of the components used to fabricate the polymer. The melting peaks for the other components like IPDI, DMPA, and BD was not observed for all samples due to the low fraction of the components in the polymer. The peaks appearing around 120 °C were due to the hard segments.

## 4. Conclusions

Various molecular weights of polyethylene glycol (PEG) (1500PEG, 4000PEG and 8000PEG) and isophorone diisocyanate were used as soft segments and hard segments respectively to produce water-soluble polyurethanes together with dimethylolpropionic acid as an emulsifier through the acetone process. Analysis was made to identify the effect of molecular weight of PEG, the ratio of PEG:IPDI, and DMPA content on the water solubility of WSPU. The results revealed that solubility of WSPU in water was dependent on the molecular weight of PEG, PEG:IPDI molar ratio, and DMPA content. As the molecular weight of PEG increased, water solubility tendency of WSPU was hindered provided that other variables held constant. An increase in the amount of hard segment (increase the ratio of IPDI), hindered the water solubility of WSPU and an increase in the DMPA content improved the tendency of water solubility of WSPU. The contact angle was dependent on the molecular weight of PEG and the amount of hydrophilic groups added (DMPA). The thermal degradation results depict that the thermal degradation temperature of WSPU increased as the average molecular weight of PEG increased.

## Figures and Tables

**Table 1:** Molecular weight, OH:IPDI molar ratio, and the water solubility of each samples.

Experiment	OH:IPDI (molar)	Contact Angle	Polyol Type	DMPA (%)	^1^HNMR	Molecular Weight (g/mol) Approximate Value	Solubility
1.	1:1	20°	1500PEG	2%			Soluble
2.	1:1	13.45°		4%		1350	Soluble
3.	1:6	20.3°		2%			Soluble
4.	1:6	17.37°		4%	38	1350	Soluble
5.	1:9	29°		2%			Soluble
6.	1:9	24.87°		4%	45	1350	Soluble
7.	1:1	58.1°	4000PEG	2%			Soluble
8.	1:1	54.5°		4%		1350	Soluble
9.	1:6	64.2°		2%			Soluble
10.	1:6	56.51°		4%	47	1350	Soluble
11.	1:9	66.3°		2%			Half soluble
12.	1:9	63.93°		4%	62	1350	Half soluble
13.	1:1	62.2°	8000PEG	2%			Half Soluble
14.	1:1	55.03°		4%		1350	Half soluble
15.	1:6	66.4°		2%			Half soluble
16.	1:6	61.88°		4%	54	1350	Half soluble
17.	1:9	67.9°		2%			Half soluble
18.	1:9	63.67°		4%	70	1350	Half soluble

^1^HNMR: the number of protons found by integration of the NMR splitting points.
